# Effectiveness of mobile SMS based counselling intervention in improving the knowledge, attitude, and practices of HIV/AIDS patients enrolled in hospitals/NGOs in Terengganu, Malaysia: a mixed mode study protocol

**DOI:** 10.1186/s12889-020-08910-3

**Published:** 2020-05-26

**Authors:** Md Mosharaf Hossain, Ruhani Binti Mat Min, Zikri Muhammad, Kulanthayan K. C. Mani

**Affiliations:** 1 Faculty of Business, Economics & Social Development, University Malaysia Terengganu, 21030 Kuala Terengganu, Malaysia; 2 Faculty of Medicine and Health Sciences, University Putra Malaysia, 43400 Serdang, Selangor Malaysia

**Keywords:** HIV/AIDS, SMS, Mobile, Counselling intervention, Malaysia

## Abstract

**Background:**

HIV/AIDS is one of the most serious problems in many parts of the world, and is a high priority for health managers and decision-makers. The aim of the qualitative part of this study will be to develop a mobile SMS (short messaging services) counselling intervention to prevent HIV/AIDS, while the quantitative part will be to test the effectiveness of a mobile SMS counselling intervention to improve the knowledge, attitude, and practices of patients concerning the prevention of HIV/AIDS.

**Method:**

A mixed methods approach will be used. Qualitative part: *Design:* focus group discussions (FGDs) will be conducted. *Setting:* Hospital/NGOs in Terengganu, Malaysia. *Participants:* Three FGDs will be conducted with male and female HIV/AIDS patients, and one group of local community leaders. One FGD will be conducted for each group. Three in-depth interviews (IDIs) will be conducted with patients who had HIV/AIDS, of which one will be female. Quantitative part: *Design:* a cluster randomized clinical trial with 384 HIV/AIDS patients in Terengganu, Malaysia. *Intervention:* Mobile SMS counselling intervention for patients concerning the prevention of HIV/AIDS.

**Results:**

The main outcomes of this study will be the differences in knowledge, attitude, and practices of patients concerning the prevention of HIV/AIDS between the baseline and immediate follow-up after the intervention, and after 3 months.

**Conclusions:**

The mobile based SMS counselling intervention developed will be effective in improving the knowledge, attitude, and practices of patients concerning HIV/AIDS prevention in Terengganu, Malaysia.

**Trial registration:**

Thai Clinical Trials Registry, TCTR20200212001; 7/02/2020.

## Background

According to the World Health Organization, approximately 35 million people worldwide are infected with HIV/AIDS and it appears that the disease will be identified as the most important cause of death in the world by 2020 [1]. In Malaysia, the country’s epidemic was mostly pushed by people who injected drugs (PWID) at an early stage, but this sample shifted towards increasing sexual transmission with the PWID/sexual transmission ratio decreasing from 4 in 2000 to 0.2 in 2015. People who inject drugs (PWID), female sex workers (FSW), transgender human beings (TG), and men who have sex with men (MSM) are identified as being the most affected widespread populations, with changes for infection exceeding 5%. The vast majority of them are above 25 years of age [2].

HIV challenges a person physically, socially, and psychologically. HIV is further recognized as a relatively stigmatized disorder [3]. In addition, it can additionally threaten a sense of meaning, purpose, and significance in life [4]. HIV causes people to become infected with physical and psychological problems [5]. In this regard, it is difficult for PLHIV to admit the truth that they are infected with HIV [6], which contributes to self-denial [7] and hinders any correction in their behaviour [8].

HIV/AIDS is among the diseases that not only affects the physical aspects, but also the social and psychological conditions of patients [9]. In addition to the physiological effects of the disease, people living with HIV/AIDS (PLWHA) may encounter numerous problems, such as discrimination, losing social status and role, changes in the patterns of relationships (intimacy), losing jobs and financial resources, and facing problems to provide the required medicines [10]. Many of these problems are also common among other people who suffer from other chronic diseases, but the stress associated with social and family problems arising from the diseases, such as social stigma and exclusion, especially by support groups, is intensely and uniquely threatening to people with HIV/AIDS [11]. Community and social network members may fear being infected with the HIV disease and they are frightened of taking care of HIV/AIDS patients [12].

HIV/AIDS is one of the diseases that currently affect not only the physical aspects, but also the social and psychological conditions of patients [9]. In addition to the physiological effects of the disease, people living with HIV/AIDS (PLWHA) may face serious problems, such as discrimination, loss of social status and roles, change in relationship patterns (intimacy), loss of work and economic resources, and problems in obtaining the required medications [10]. Many of these problems are more frequent than those faced by other people who are struggling with other chronic diseases, as it is not just the ailment, it is the stress associated with social and home troubles, such as social stigma and exclusion in particular, as some of those providing assistance and help groups are intensely and exceptionally threatened by people with HIV/AIDS [11]. Community and social network participants may fear that they will be infected with HIV, other ailments, and are afraid of caring for patients with HIV/AIDS [12].

However, there has been a lack of research on the challenges of life among people affected with HIV (PAWH) based on the mode of HIV transmission, and, hence, this study examines the different experiences and challenges of life among the participants based on the mode of transmission. The aim of this research is a trial to test the efficacy of a MOBILE SMS based counselling intervention for the prevention of HIV/AIDS in Malaysia.

## Methods/design

The main objective of this study is to develop, implement, and assess the effectiveness of the mobile SMS counselling intervention to improve the knowledge, attitude, and practices of HIV/AIDS patients concerning HIV/AIDS prevention in Malaysia. The specific objectives are given below: to assess the patients initial response to mobile SMS counselling intervention in terms of acceptability, feasibility, and sustainability; to develop mobile SMS counselling intervention for HIV/AIDS prevention; to identify factors associated with the willingness to read text messages on HIV/AIDS prevention in Malaysia; to determine the socio-demographic and environmental factors, level of knowledge, attitude, and practices of patients on HIV/AIDS prevention; to develop and implement a Mobile SMS counselling HIV/AIDS prevention intervention to improve the knowledge, attitude, and practices of HIV/AIDS patients; to determine the association among the socio-demographic factors and environmental factors with knowledge, attitude, and practices of HIV/AIDS patients about HIV/AIDS prevention at the baseline and post intervention; to compare the mean scores for knowledge, attitude, and practices among HIV/AIDS patients between the intervention and the control group at the baseline and post intervention (within group and between groups); and to evaluate the effectiveness of Mobile SMS counselling intervention among HIV/AIDS patients to improve the knowledge, attitude, and practices concerning HIV/AIDS.

The hypotheses are given below: There is an association between the willingness to read text messages and HIV/AIDS patients in Malaysia; there is no significance in the socio-demographic and environmental factors, level of knowledge, attitude, and practice of patients concerning HIV/AIDS prevention; there is an association between the social-demographic and environmental factors, and the knowledge, attitude, and practices of HIV/AIDS patients concerning HIV/AIDS prevention at baseline and post-intervention; there is a significant difference in the mean scores for the knowledge, attitude, and practices of HIV/AIDS patients between the intervention and control group at the baseline and post-intervention (within and between groups), and the application of Mobile SMS counselling intervention to improve the impact of the HIV/AIDS prevention knowledge, attitude, and practices among patients, between the intervention and the control group.

The research questions are given below: What are the socio-demographic and environmental factors associated with the knowledge, attitude, and practice of HIV/AIDS patients? What is the difference in the mean scores for the knowledge, attitude, and practices of HIV/AIDS patients concerning HIV/AIDS prevention for the intervention and control groups? And what is the impact of Mobile SMS counselling intervention on the knowledge, attitude, and practices of HIV/AIDS patients?

**Fig. 1:**
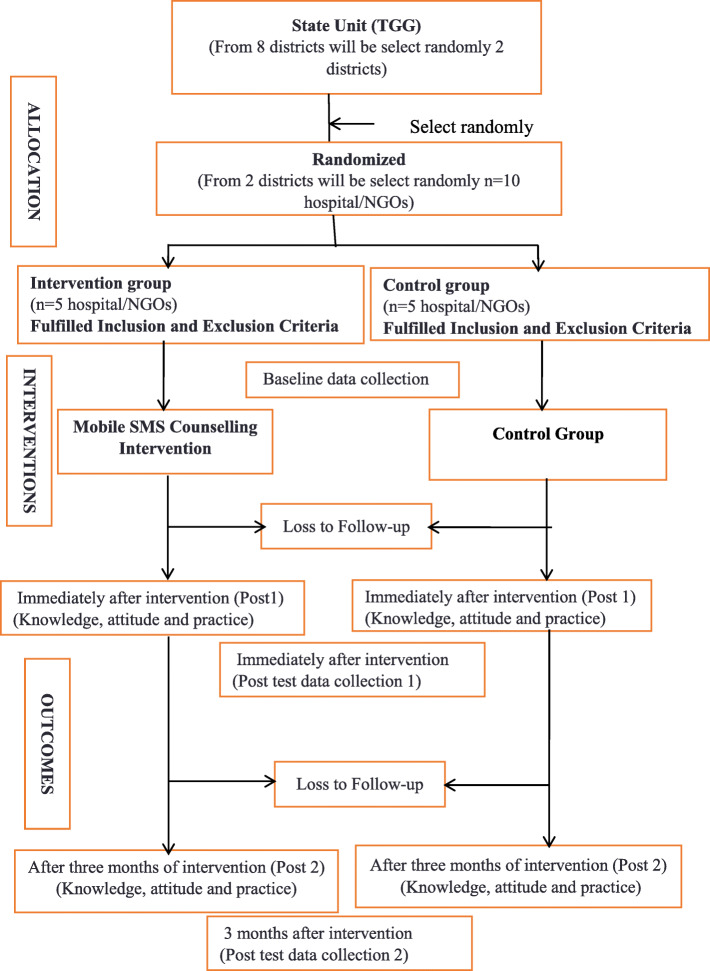
Study flow diagram showing the key components of the intervention

This is a mixed methods research. The first phase will be a qualitative study to develop a mobile SMS counselling intervention concerning the knowledge, attitude, and practices of patients concerning HIV/AIDS prevention, while the second phase will be a quantitative study to implement and evaluate the effectiveness of a mobile-based counselling intervention concerning the knowledge, attitude, and practices of patients in respect of HIV/AIDS prevention. Three focus group discussions (FGDs) will be conducted with 6 male HIV/AIDS patients with FGDs, 6 female HIV/AIDS patients with FGDs, 10 community leaders from a village with FGDs, and three in-depth interviews (IDIs) will be conducted with 3 HIV/AIDS patients. The second phase, the cluster randomized clinical trial, will be carried out for approximately 12 months from September 2019 to July 2020. The hospital/NGOs (or cluster) will be the unit of randomization, and an equal number of hospitals/NGOs will be randomized to one of the two arms. This phase includes a Community trial and pre- and post-assessment through face-to-face interviews using a semi-structured questionnaire (Fig. 1). The study population will be HIV/AIDS patients in the Kuala Terengganu and Kuala Nerus districts of Malaysia. A complete list of HIV/AIDS patient registration will be collected from the Hospitals/NGOs in Kuala Terengganu and Kuala Nerus districts from January 2018 to March 2019. The sampling units will be individual patients who are admitted into the Hospital/NGOs between January 2018 and March 2019 and who are eligible according to the inclusion criteria for this study. The criteria for inclusion in this study include all persons diagnosed with HIV ≥ 18 years of age. Patients excluded from this study are those patients who are HIV positive but diagnosed with psychiatric disorders and are mentally incapacitated. The sample size estimation formula for hypothesis testing of the two-group comparison is used [13]:

$$ \mathrm{N}={\left\{\mathrm{Z}1-\upalpha /2\ \sqrt{2}\tilde{\mathrm{P}}\left(1-\tilde{\mathrm{P}}\right)+\mathrm{Z}1-\upbeta \sqrt{\mathrm{P}}1\left(1-\mathrm{P}1\right)+\mathrm{P}2\left(1-\mathrm{P}2\right)\right\}}^2/{\left(\mathrm{P}1-\mathrm{P}2\right)}^2 $$

### N = 87

Where P1 = HIV Prevalence in 2012 = 0.189 & P2 = HIV Prevalence in 2014 = 0.163 [2]. P̃ = (P1 + P2)/2 = 0.176. Z1 – α/2 = standard error when α = 0.05 (95% confidence interval) = 1.96, and Z1 – β = standard error associated with power = 0.842 (β = 0.20) and Power (1 – β) = 80%.

As participants are nested within in villages, a potential design effect needs to be considered. The assumed number of participants per hospital/NGOs is 20, and an intra-cluster correlation coefficient of 0.05 can be expected. This would result in a design effect = 1 + (m-1) × ICC = 1 + (20–1) × 0.05 = 2. Based on the above formula, the required minimum sample size for each group, n is **174.** To factorise in 10% attrition (32), the total required per group is **192**; hence, a total of **384** patients will be required for the sample in both groups.

**Fig. 2:**
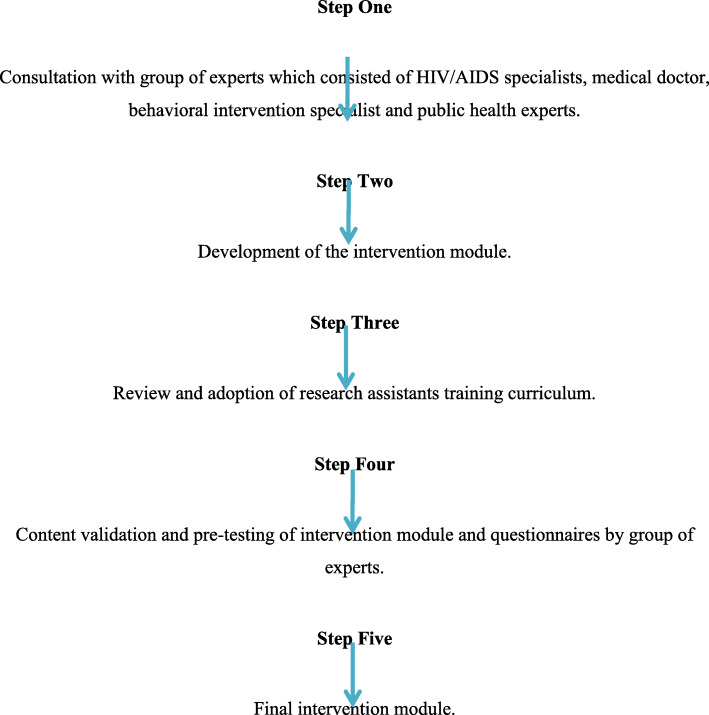
Schematic diagram of the development of the intervention module

Figure 2 below shows a schematic diagram for the development of the intervention module used in this study. The module will be developed through the process of consultation with a group of experts who will include one professor of statistics, one expert in behavioural intervention, one senior medical doctor, and one HIV/AIDS specialist (Fig. 2). The mobile based SMS counselling intervention aims to increase the knowledge, attitude, and practices of patients concerning HIV/AIDS prevention in Malaysia. The SMS will be developed by the researcher based on FGDs and a literature review. It uses informal language and will be sent weekly on Friday, which is a holiday. These factors have been shown to have an impact and positively affect the message acceptability when using SMSs for sexual health promotion [14, 15]. Mobile phones and the Internet are popular everywhere in Malaysia, and are well-liked by people, so the study aims to determine whether they would be effective and improve patient’s knowledge, attitude, and practices concerning HIV/AIDS prevention.

The control group patients will not receive any SMS during the study periods, but the usual information received from the mass media (newspaper, TV, Radio) or any campaign for HIV/AIDS prevention. However, they will receive the SMS counselling intervention about HIV/AIDS after data collection.

The Statistical Package for Social Science (SPSS) version 22 will be used to analyse the data. Data will be collected, checked, cleaned, and data entry will be conducted carefully and successfully. Factors with a statistically significant association with the socio-demographic and environmental variables on HIV/AIDS prevention knowledge, attitude, and practices will be identified at the baseline, immediate post intervention, and after 3 months follow up in the logistic model (ρ-value of 0.05) controlling for other variables. To determine the significant difference between the intervention and the control groups, a t-test and chi-square test will be used. To identify the impact of two different interventions, 2-way repeated measure ANOVA and one-way ANOVA will be used.

The main findings are given below: development of mobile SMS based counselling intervention for prevention of HIV/AIDS with FGDs; identification of implementation process for mobile SMS based counselling intervention for prevention of HIV/AIDS patients; development of tools for measuring the effectiveness of Mobile SMS based counselling intervention for prevention of HIV/AIDS patients; development of an effective intervention for HIV/AIDS patients in a low resource setting to improve the knowledge, attitude, and practices concerning HIV/AIDS prevention, and assessment of the effectiveness of a mobile SMS based counselling intervention programme on the HIV/AIDS prevention knowledge, attitude, and practices of HIV/AIDS patients.

## Discussion

This paper constitutes a protocol for the effectiveness of SMS mobile based counselling intervention in a developing country. The mobile based SMS counselling intervention on patients affected by HIV/AIDS is the first controlled trial testing. Most cases of HIV/AIDS patients go unrecorded due to the high stigma, which is one of the limitations of this study. The evidence generated will contribute significantly by providing evidence concerning the use and effectiveness to influence decisions. It will provide an attractive, cost-effective model to increase the knowledge and awareness of HIV/AIDS and reduce HIV/AIDS in Malaysia, given that the mobile based SMS counselling intervention is effective. The programme could be easily disseminated to other middle/low-income countries nationwide, or through national or international prevention campaigns.

## Data Availability

The datasets generated and/or analysed during the current study are not publicly available but are available from the corresponding author on reasonable request.

## Abbreviations

ANOVA: Analysis of variance
FGDs: Focus group discussion
HIV/AIDS: Human immunodeficiency virus/ Acquired immunodeficiency syndrome
IDIs: In-depth interviews
MSM: Men who have sex with men
PLWHA: People living with HIV/AIDS
PWID: People who injected drugs
SMS: Short messaging services
TG: Transgender human beings
