# Correction to: Effectiveness of mobile SMS based counselling intervention in improving knowledge, attitude and practices of HIV/AIDS patients enrolled in hospitals/NGOs in Terengganu, Malaysia: a mixed mode study protocol

**DOI:** 10.1186/s12889-020-09395-w

**Published:** 2020-08-24

**Authors:** Md Mosharaf Hossain, Ruhani Mat Min, Zikri Muhammad, Kulanthayan K. C. Mani

**Affiliations:** 1 Faculty of Business, Economics & Social Development, Universiti Malaysia Terengganu, 21030 Kuala Terengganu, Malaysia; 2 Faculty of Medicine and Health Sciences, Universiti Putra Malaysia, 43400 Serdang, Selangor Malaysia

**Correction to: BMC Public Health 20, 787 (2020)**

**https://doi.org/10.1186/s12889-020-08910-3**

Following publication of the original article [1], the authors identified an error in the author name of Ruhani Binti Mat Min.

The incorrect author name is: Ruhani Binti Mat Min

The correct author name is: Ruhani Mat Min

The author group has been updated above/

It was also highlighted that the original article [1] contained an error in the Acknowledgement and funding section.

This Correction article shows the correct Acknowledgment section and Funding section.

**Acknowledgments**

We would like to gratefully acknowledge the Universiti Malaysia Terengganu (UMT) and Ministry of Education, Malaysia for their grants on our manuscript.

**Funding**

The study was supported by Ministry of Education, Malaysian Government (**Grant No. 59594**) and Universiti Malaysia Terengganu and the study protocol underwent peer review by the funding body and Ethical committee of Universiti Malaysia Terengganu. The funders had no role in study design, data collection and analysis, decision to publish, or preparation of the manuscript.
